# Sevoflurane and Desflurane Spin–Decoherence Effect on Fe(III)acetylacetonate Redox Process

**DOI:** 10.3390/molecules30224341

**Published:** 2025-11-10

**Authors:** Neha Kumari, Andrea Severini, Mauro Borghi, Monica Montecchi, Luca Pasquali, Elena Colombini, Gabriele Melegari, Alberto Barbieri, Enrico Giuliani, Massimo Innocenti, Fabrizio Roncaglia, Tapan Das Kumar, Claudio Fontanesi

**Affiliations:** 1 Department of Engineering “Enzo Ferrari”, (DIEF), University of Modena, Via Vivarelli 10, 41125 Modena, Italy; nehasingh121998@gmail.com (N.K.); monica.montecchi@unimore.it (M.M.); luca.pasquali@unimore.it (L.P.); elena.colombini@unimore.it (E.C.); 2 Department of Chemical and Geological Science, (DSCG), University of Modena, Via Campi 103, 41125 Modena, Italy; andrea.severini@unimore.it (A.S.); fabrizio.roncaglia@unimore.it (F.R.); 3 Institute for Microelectronics (TU Wien), Gusshausstrasse 27–29, 1040 Vienna, Austria; mauro.borghi@unimore.it; 4 Department of Physics, University of Johannesburg, P.O. Box 524, Auckland Park 2006, South Africa; 5 IOM-CNR, Strada Statale 14, Km. 163.5 in AREA Science Park, Basovizza, 34149 Trieste, Italy; 6 Anaesthesia and Intensive Care, Azienda Ospedaliero Universitaria Modena, Via del Pozzo 71, 41211 Modena, Italy; gabriele.melegari@unimore.it (G.M.); alberto.barbieri@unimore.it (A.B.); 7 NEURONGUARD, Via Ludovico Castelvetro, 19, 41124 Modena, Italy; enrico.giuliani@neuronguard.com; 8 Department of Chemistry “Ugo Schiff”, (DICU), University of Firenze, Via Della Lastruccia, 41125 Sesto Fiorentino, Italy; massimo.innocenti@unifi.it; 9 National Interuniversity Consortium of Materials Science and Technology (INSTM), Via G. Giusti 9, 50121 Firenze, Italy; 10 Interdepartmental Centre H2-MORE, University of Modena and Reggio Emilia, Via Università 4, 41125 Modena, Italy; 11 Department of Chemical and Biological Physics, Weizmann Institute of Science, Rehovot 7610001, Israel; tapan-kumar.das@weizmann.ac.il

**Keywords:** anesthesia, desflurane, sevoflurane, iron(III) acetylacetonate, spin, CISS

## Abstract

This study investigates the influence of sevoflurane and desflurane on the electrochemical behavior of the Fe(III)-acetylacetonate (Fe(acac)_3_) complex. Using cyclic voltammetry (CV), we demonstrate that while Fe(acac)_3_ exhibits reversible redox behavior in an oxygen-free environment, the presence of dissolved oxygen renders the system irreversible, leading to the formation of a thick, reddish film on the electrode surface upon potential cycling. Notably, the addition of sevoflurane and desflurane restores the electrochemical reversibility and dramatically inhibits this film formation. Raman spectroscopy of the resulting films confirmed structural changes which are consistent with this inhibiting action. Furthermore, X-ray photoelectron spectroscopy (XPS) analysis reveals that the iron in the film remains predominantly in the Fe^3+^ oxidation state even after prolonged electrochemical reduction cycles. These findings suggest that the anesthetics act by inhibiting the interaction between the Fe(acac)_3_ complex and oxygen, likely through a spin–decoherence mechanism. This work highlights the critical role of anesthetics in modifying the electrochemical behavior of metal-oxygen complexes, with potential implications for sensing, electrocatalysis, and bio-oriented systems.

## 1. Introduction

Acetylacetonate (acac) complexes, particularly those of transition metals, are a significant class of coordination compounds widely investigated for their diverse redox and catalytic properties. The electrochemical behavior of the Fe(acac)_3_ redox couple has attracted extensive research due to its utility in diverse applications such as electrocatalysis, sensing technologies, and fundamental studies of electron transfer mechanisms [1].

As Fe(acac)_3_ exhibits reversible redox activity that is exceptionally sensitive to its surrounding chemical environment, it is an ideal candidate for studying external molecular influences on electrochemical processes.

**Figure 1:**
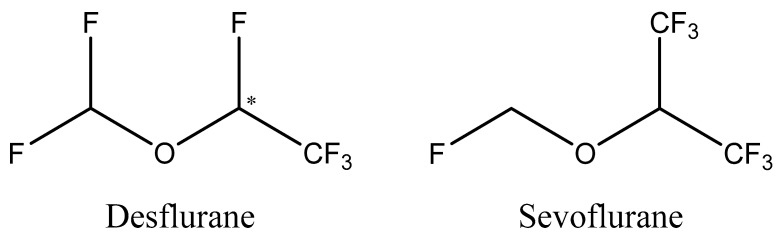
Chemical structures of desflurane and sevoflurane.

Previous studies have largely explored the interaction of Fe(acac)_3_ complexes with common ligands and solvents, characterizing their fundamental redox activity and stability under various electrochemical conditions [2]. However, limited research has explicitly investigated the interactions between Fe(acac)_3_ and biologically relevant molecules, such as halogenated anesthetics, despite their widespread clinical use and potential environmental and biological impacts. Halogenated anesthetics including sevoflurane (C_4_H_3_F_7_O) and desflurane (C_3_H_2_F_6_O), Figure 1, are commonly utilized in medical procedures due to their rapid onset, minimal metabolism, and favorable pharmacokinetics.

Their unique chemical structure features multiple fluorinated functional groups that could interact with transition metal centers, potentially altering redox kinetics and electronic properties [3,4]. Such interactions may involve weak non-covalent interactions, like halogen or hydrogen bonding, that are known to modulate electron density and exert ligand-field effects. These variations ultimately affects redox behavior [5,6,7,8,9]. Recent investigations have explored the influence of small molecules on the electron transfer mechanism of Fe(acac)_3_, particularly in the presence of dissolved gases such as oxygen [8,9,10,11]. Still the role of volatile fluorinated anesthetics in modulating redox-active systems is still an unsolved conundrum. The fundamental role of electron spin in biological systems was demonstrated in seminal papers by Pauling on the magnetic properties of hemin, related to the spin-multiplicity of iron [12,13,14]. Recent works focused on the oxygen reduction reaction (ORR) highlighted the crucial role of electron spin in both cellular respiration and the mechanism of anesthesia. The ORR, the final step in respiration, is a spin-forbidden process where triplet-state oxygen must be converted to singlet-state products. This reaction is typically made possible by enzymes with heavy atoms or metal cofactors that possess strong spin–orbit coupling, which can “mix” spin states to enable the reaction [9,10]. Building on this understanding, researchers have proposed that general anesthetics act by disrupting these fundamental spin dynamics. A seminal study by Turin and colleagues demonstrated that general anesthetics cause rapid changes in electron spin, linking these quantum effects to the well-known Meyer–Overton relationship and suggesting a fundamental role for spin in anesthesia [15]. Subsequent research by Naaman and co-workers has further explored the specific mechanisms, revealing that the efficiency of ORR is enhanced by increasing spin coherence in the electron transfer: it is shown that a loss of spin coherence leads to a significant decrease in ORR efficiency, suggesting a potential explanation for how anesthetics hinder respiration [10]. In a related paper, it was also demonstrated that chirality can modulate ORR efficiency through the chiral-induced spin selectivity (CISS) effect, which controls the spin alignment of electrons [8]. Together, these studies suggest that by inducing spin decoherence, anesthetics can disrupt the crucial final step of cellular respiration, providing a compelling hypothesis for their effects at a cellular level. The connection between our work and physiological activity lies in the role of hemoglobin (Hb), which is responsible for transporting oxygen from the lungs to tissues. Hb iron in the deoxygenated state is high-spin Fe^2+^(paramagnetic) and becomes low-spin upon O_2_ binding. Inhaled anesthetics like sevoflurane and desflurane are known to influence both O_2_ utilization in tissues and the Hb–O_2_ binding dynamics. These anesthetics also suppress overall respiration and metabolic O_2_ consumption in vivo by reducing the cerebral metabolic rate of O_2_ and myocardial oxygen demand, they effectively lower the amount of O_2_ being utilized by tissues [7]. In summary, sevoflurane and desflurane not only alter hemoglobin–O_2_ affinity but also suppress respiratory function and tissue O_2_ utilization, a combined effect that modifies the body’s overall oxygen dynamics [7,16]. Within this picture, this manuscript addresses this knowledge gap by exploring the influence of sevoflurane and desflurane on the electrochemistry of Fe(acac)_3_ in a non-aqueous medium. Our work serves as a potential model system for understanding how halogenated anesthetics can modulate oxygen interactions with metal centers. We hypothesize that these anesthetics modulate the redox behavior of the complex by altering its local environment and inducing spin decoherence, a phenomenon with important implications for electron transfer efficiency [16,17,18]. Our research strategy bridges electrochemical and spectroscopic analyses to provide a comprehensive understanding of these interactions at both the molecular and macroscopic levels [19]. We demonstrate that the presence of these anesthetics leads to measurable changes in the Fe(acac)_3_ cyclic voltammetry response, including unique current enhancements and potential shifts that correspond to oxygen reduction and the possible formation of Fe(II)-O_2_ or Fe(IV)=O species [20,21]. We also observed the formation of a dark brown film upon extended potential cycling, a phenomenon of possible electropolymerization not previously reported for the Fe(acac)_3_ compound [15]. Using Raman spectroscopy, we validate these macroscopic observations by providing vibrational evidence of altered ligand field environments [3,4]. This integrated approach, which explicitly explores anesthetic-induced changes in electropolymerization, differentiates our work from earlier studies [19] and positions our findings as highly relevant to biomedical sensing and chemical neurobiology.

## 2. Results and Discussion

### 2.1. Cyclic Voltammetry Analysis

#### 2.1.1. Influence of Dissolved Oxygen

The electrochemical behavior of Fe(acac)_3_ was investigated using CV in a potential range up to −1.6 V. All experiments were performed with a 0.1 M TBATFB in MeCN electrolyte and a scan rate of 50 mV/s or 100 mV/s.

**Figure 2:**
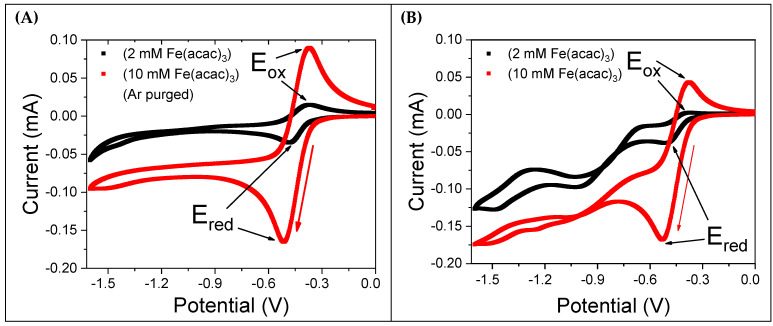
CV curves of Fe(acac)_3_ dissolved in a solution containing 0.1 M TBATFB in MeCN. A gold working electrode, platinum counter electrode, and Ag/AgCl/KCl_sat_ reference electrode were used. The potential scan rate was 50 mV/s, as indicated by the red arrow. (**A**) CVs of 2 mM and 10 mM Fe(acac)_3_ solutions after degassing with Ar for 15 min. (**B**) CVs of the same solutions in equilibrium with molecular oxygen at room temperature.

Figure 2A shows CV curves for 2 mM and 10 mM Fe(acac)_3_ solutions after degassing with argon (Ar) for 15 min. The voltammograms exhibit a quasi-reversible nature, with a peak-to-peak potential difference ($∆E\;=E_{red}-E_{ox})$ of 93 mV (detailed results are reported in Table 1). Both the forward reduction and backward oxidation current wave are nearly symmetrical. In contrast, Figure 2B presents CVs recorded in the presence of saturated molecular oxygen (2.42 mM at 298 K [22]) without Ar purging. These CVs show an irregular pattern, with the backward oxidation current being significantly smaller than the forward reduction current. Notably, the 2 mM Fe(acac)_3_ CV lacks a complete backward oxidation peak. Additionally, for potential values more negative than −0.7 V, the CVs feature additional current peaks, indicating that further redox processes are active. The significant differences observed between the CVs recorded with and without dissolved oxygen suggest that oxygen is not an inert species but plays a substantial role in the reduction in Fe(acac)_3_, likely by forming an intermediate with the reduced Fe(acac)_3_ molecular species.

#### 2.1.2. Influence of Anesthetics

**Figure 3:**
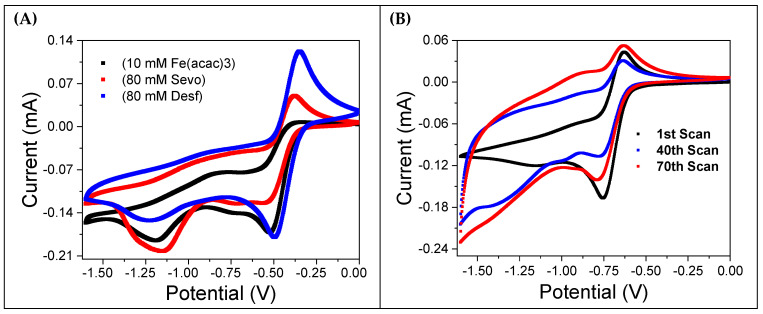
Cyclic voltammetry (CV) analysis of the influence of halogenated anesthetics on a 10 mM Fe(acac)3 solution. The solution was prepared in MeCN with 0.1 M TBATFB and was saturated with oxygen at room temperature. (**A**) CV curves comparing the Fe(acac)3 solution alone (black curve) with the addition of 80 mM sevoflurane (red curve) and 80 mM desflurane (blue curve). (**B**) The evolution of the CV pattern during repeated cycling, showing the changes in electrochemical behavior over 70 scans.

Figure 3A shows CVs for a 10 mM Fe(acac)_3_ solution saturated with oxygen. The black curve represents the solution without anesthetics, while the red and blue curves correspond to the addition of 80 mM sevoflurane and desflurane, respectively. Notably, the introduction of anesthetics restores the reversibility of the oxidation peak, which is almost entirely absent in the curve without them. This effect is more evident in the presence of desflurane. Figure 3B displays the results of recursive potential cycling in the same 10 mM Fe(acac)_3_ solution saturated with oxygen, but without anesthetics. The CVs become progressively wider with each cycle, indicating an increasing current. This is consistent with the formation of a thick reddish coating observed on the working electrode’s surface, a clear sign of electropolymerization.

The addition of sevoflurane and desflurane partly inhibits the formation of this reddish film, suggesting that the anesthetics interfere with the interaction between oxygen and the Fe(acac)_3_ complex. This behavior mirrors the results observed when the solution is degassed with argon (Figure 2A).

Overall, the tight comparison between the electrochemical results obtained from degassed solutions and those containing anesthetics strongly suggests that sevoflurane and desflurane inhibit the oxygen reduction reaction and its subsequent interaction with the Fe(acac)_3_ complex. This evidence is in good agreement with previously published work by Ron Naaman and co-workers [9,10]. A detailed and systematic study of the effects of varying Fe(acac)_3_ and anesthetic concentrations, as well as the influence of purging with argon, can be found in the Supporting Information (Sections S1 and S2, Figures S1–S5).

### 2.2. Raman and XPS Spectra of the Film Formed upon CV Cycling

**Figure 4:**
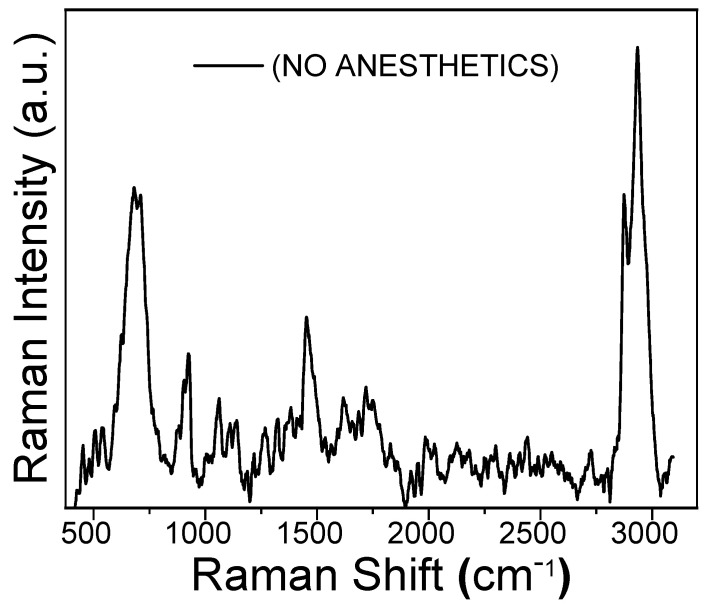
Raman spectrum of the polymeric film obtained by the electropolymerization of 10 mM Fe(acac)_3_ in oxygen-saturated acetonitrile (MeCN). The film was formed on a gold working electrode by sweeping the potential from 0.0 to −1.6 V at 50 mV/s for 68 cyclic voltammetry (CV) cycles.

Figure 4 shows the Raman spectrum of the reddish coating obtained on the gold working electrode after 68 CV cycles in the 0 to −1.6 V potential range. This film, whose formation corresponds to the CV behavior shown in Figure 3B, was obtained from a 10 mM Fe(acac)_3_ solution with oxygen present. Two prominent Raman peaks are observed at 2930 cm^−1^ and 670 cm^−1^. These are assigned to the carbon-hydrogen stretching of the methyl groups and to iron oxide, respectively. The peak at 670 cm^−1^ suggests that the iron oxide is present as a mixture of Magnetite and Wüstite [23].

Additionally, three broader and more shallow peaks are observed at approximately 900 cm^−1^, 1400 cm^−1^, and 1700 cm^−1^, which are assigned to the acetylacetonate carbonylic and five-carbon-chain vibrational modes.

The presence of the peak at 670 cm^−1^ is a key finding, as it provides spectroscopic evidence that the Fe(acac)_3_ complex decomposes during reduction in the presence of oxygen, leading to the formation of iron oxides.

**Figure 5:**
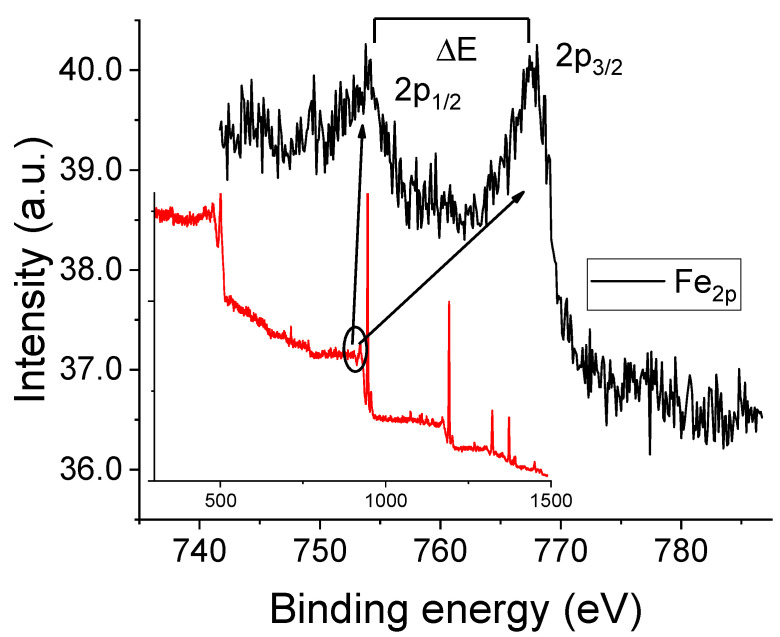
XPS spectrum of the reddish film formed on the working electrode surface after 68 CV cycles. The main figure presents the high-resolution Fe 2p core level spectrum (black curve), which was acquired from the energy interval indicated by the solid black line in the inset. The inset also shows the full survey scan of the sample (red curve).

Figures S6 and S7 show the Raman spectra obtained from the working electrode surface after 68 CV cycles with 10 mM Fe(acac)_3_ solutions containing either sevoflurane or desflurane. For comparison, the spectrum of the film formed in the absence of anesthetics is also shown (black trace) with prominent bands at approximately 460 cm^−1^ (Fe-O stretching), 1560 cm^−1^ (C=C stretching), and 2970 cm^−1^ (C-H stretching). Upon the addition of sevoflurane (red spectrum), significant changes are observed. Fe-O and C=C vibrational bands intensity decreases, while the C-H stretching peak becomes more pronounced. These changes suggest a modified structural organization induced by sevoflurane, possibly through weak halogen-bond interactions or solvation effects that alter the electron density around the metal center [1]. In contrast, desflurane (blue spectrum) causes an even greater attenuation of the Fe-O and C=C vibrational intensities. The resulting spectrum shows broader, more shallow peaks, indicating a more amorphous polymer structure and potentially stronger electron-withdrawing effects [18]. The observed Raman spectral changes suggest that both anesthetics influence the polymerization mechanism and molecular structure through weak coordination or hydrogen bonding interactions [24]. Figure 5 displays the XPS results for the thick reddish film obtained after CV cycling in the presence of oxygen (corresponding to the conditions of Figure 3B). The Fe2p spectral region exhibits a doublet structure resulting from spin–orbit splitting between the 2p_1_/_2_ and 2p_3_/_2_ electronic states. The presence of characteristic satellite features in X-ray photoelectron spectra is a common method for determining the valence state of iron [25]. For instance, hematite (Fe_2_O_3_) shows a binding energy difference, ΔE, of approximately 8.5 eV between the main 2p_3_/_2_ peak and its satellite, while for FeO, this difference is around 6.0 eV [25]. In our sample, the Fe 2p_3_/_2_ and Fe 2p_1_/_2_ peaks are observed at approximately 711.0 eV and 724.7 eV, respectively. The presence of two satellite peaks, centered at about 719.4 eV and 733.3 eV, strongly suggests that the iron in the film is in the Fe(III) oxidation state. This conclusion is in tight agreement with the Raman results, specifically the prominent peak observed at 670 cm^−1^, which was assigned to iron oxides [25,26]. XPS spectra for other experimental conditions (Ar-purged and anesthetic-containing solutions) are provided in the Supporting Information.

## 3. Spin Mediated ORR-Anesthetics Interaction

### 3.1. Electrochemical Mechanism

To establish a baseline, we first investigated the electrochemical behavior of the blank electrolyte solution (0.1 M TBATFB in MeCN) under both oxygen and argon atmospheres. As shown in Figure S1, a negligible current response was recorded for the solution purged with argon, confirming the electrochemical inertness of the base electrolyte in the absence of oxygen. Conversely, under an oxygen atmosphere, two distinct cathodic peaks were observed. The first, at approximately −0.83 V vs. Ag/AgCl (−0.16 mA), corresponds to the one-electron reduction in molecular oxygen (O_2_) to the superoxide radical (O_2_•^−^). The second, at approximately −1.42 V (−0.18 mA), is attributable to the further reduction in the superoxide radical to a peroxide species (O_2_^2−^).

The electrochemical reduction of oxygen can be described by the following two-step mechanism:

(1) $$O_{2}+e^{-}\to O_{2}^{•-}$$

(2) $$O_{2}^{•-}+e^{-}\to O_{2}^{2-}$$

Both the peak positions and currents match the results reported by Costentin and Savéant for the O_2_/superoxide couple in acetonitrile under similar experimental conditions [1].

### 3.2. Concentration-Dependent Electrochemical Activity of Fe(acac)_3_

As illustrated in Figure 2A,B, Figures S2 and S3, the electrochemical behavior of Fe(acac)_3_ was investigated at concentrations of 2 mM, 10 mM, and 100 mM in a 0.1 M TBATFB/MeCN electrolyte. The voltammograms show well-defined redox peaks at approximately −0.65 V and −0.3 V vs. Ag/AgCl. The peak current is directly proportional to the Fe(acac)_3_ concentration, corresponding to the reduction in Fe(III) to Fe(II) in the forward scan and the reverse oxidation in the backward scan.

In the presence of oxygen, an additional negative current peak is observed between −0.8 V and −1.6 V. Those peaks are indicative of a catalytic reduction in dissolved oxygen by the electrochemically generated Fe(II) species, which is formed during the cathodic scan. This process leads to the formation of a low-stability iron-oxo intermediate, ultimately resulting in the deposition of a thick reddish film on the electrode surface. The formation of this film is consistent with the behavior shown in Figure S1, and a possible reaction scheme is described by reactions (3) and (4):

(3) $$Fe{\left(acac\right)}_{3}+e^{-}\to {Fe(acac)}_{2}^{-}+{acac}^{-}$$

(4) $${Fe(acac)}_{2}^{-}+O_{2}^{•-}(\mathrm{or}+O_{2}^{2-})\;\to {Fe(acac)}_{2}^{-}\cdots (O)\;{\left(\mathrm{iron-oxo}\;\mathrm{intermediates}\right)}^{‡}$$

^‡^ Formation of a thick film. Magnetite and Wüstite is observed, as suggested by Raman results.

### 3.3. Thick Film Formation Under Repeated Cycling

Repeated CV scans of the 10 mM Fe(acac)_3_ solution in the presence of oxygen, as shown in Figure 3B, Figures S4 and S6, reveal a progressive change in the current for potentials more negative than −0.7 V. Over successive scans (10th to 68th), a significant increase in current magnitude is observed, indicating a progressive electrochemical activation of the electrode surface. This behavior highlights the dynamic nature of the Fe(acac)_3_ electrochemical system under repeated cycling, with the formation of a thick reddish film on the gold electrode surface. This film results from prolonged electrochemical reduction and is consistent with the proposed reactions (3) and (4) [9]. It should be emphasized that the formation of this thick reddish film is observed only in solutions free from anesthetics.

### 3.4. Influence of Sevoflurane and Desflurane on the Electrochemical Behavior of Fe(acac)_3_

The addition of sevoflurane or desflurane significantly alters the voltammetric response. With increasing anesthetic concentration (from 20 mM, 40 mM, to 80 mM), a systematic increase in the backward oxidation current peak is observed around −0.3 V (Figure 3A). This evidence demonstrates that both sevoflurane and desflurane inhibit the Fe(acac)_3_/oxygen interaction, which in turn modifies the electrode surface dynamics. The macroscopic result is a significant reduction in the electrode coating. The film formation is almost negligible even after more than 100 scans. A qualitative, visual comparison of the electrode surfaces after 68 CV cycles, both with and without anesthetics, is provided in Figure S6.

## 4. Materials and Methods

### 4.1. Chemicals

Reagent grade tetrabutylammonium tetrafluoroborate (TBATFB, ≥99%), tetrabutylammonium perchlorate (TBAP ≥ 99%), and iron(III)acetylacetonate (Fe(acac)_3_, ≥98.5%) were purchased from Sigma Aldrich (Milano, Italy). Anhydrous acetonitrile (MeCN, HPLC grade, ≥99.9%) was purchased from Carlo Erba Reagents (Cornaredo (MI), Italy). Medical-grade sevoflurane and desflurane anesthetics (≥99.5%) were obtained from commercial medical suppliers, Baxter (Sesto, Fiorentino (FI), Italy).

### 4.2. Electrochemical Experiments

Electrochemical experiments were performed using a PGSTAT 128N Autolab Metrohm (Origgio (VA), Italy), potentiostat–galvanostat. A conventional three-electrode setup was employed, consisting of a gold rod working electrode (WE, 3 mm diameter), a platinum wire counter electrode (CE, 0.50 mm diameter, 4 cm length), and an Ag/AgCl/KCl_sat_ reference electrode (RE). All potentials are reported with respect to the Ag/AgCl/KCl_sat_ reference electrode. All solutions contained 0.1 M tetrabutylammonium tetrafluoroborate TBATFB as the electrolyte in MeCN, unless otherwise specified. Cyclic voltammetry (CV) studies were conducted on Fe(acac)_3_ solutions at concentrations of 2 mM, 10 mM, and 100 mM. The influence of halogenated anesthetics was investigated by adding sevoflurane and desflurane to 10 mM Fe(acac)_3_ solutions at concentrations ranging from 20 mM to 80 mM. To investigate the interaction between Fe(acac)_3_ and dissolved oxygen, CV measurements were recorded both with and without gas purging, in the presence and absence of anesthetics. All experiments were performed at ambient laboratory conditions and without stirring. Data acquisition and analysis were performed using NOVA 2.1 software supplied with the Autolab potentiostat–galvanostat system.

### 4.3. X-Ray Photoelectron Spectroscopy (XPS)

Photoemission spectra were acquired using an Omicron EA125 electron analyzer (OMICRON Technologies Italia Srl, Vimercate (MI) Italy), at normal emission. XPS was performed with non-monochromatic Al *K_α_* photons (*hν =* 1486.6 eV) from a Vacuum Generators XR3 dual anode source operated at 15 kV, 18 mA. Spectral resolution was set to 1.0 eV for wide overview scans and 0.5 eV for detailed single-peak analysis.

### 4.4. Raman Spectroscopy

Raman spectra were collected using a Horiba Raman LabRAM HR Evolution Microscope (Horiba Italia, Roma (RM), Italy). The system utilized a 532 nm Nd-Yag laser with a maximum power of 1 mW for excitation. Spectra were acquired using a 600 g/mm or 1800 g/mm diffraction grating. The instrument was equipped with an Olympus BXFM-ILHS optical microscope and features a confocal pinhole spot of 0.005 mm (Olympus Italia, Roma (RM), Italy), and a front illuminated CCD detector featuring an open electrode multi pin phased, spectral range 200–1100 nm, with multiple Peltier cooling system, 1024 × 256 × 16 matrix.

## 5. Conclusions

Our electrochemical and spectroscopic investigations provide direct evidence of the influence of halogenated anesthetics on the Fe(acac)_3_ redox system. We have shown that prolonged cyclic voltammetry in the presence of oxygen leads to the formation of a thick, reddish coating on the electrode surface. Both XPS and Raman spectroscopy confirm that this film is primarily composed of iron oxides, likely a mixture of Magnetite and Wüstite. A key finding is that the presence of sevoflurane and desflurane in the solution dramatically inhibits the formation of this coating, even when oxygen is present. This result mirrors the behavior observed when the solution is degassed with argon. This evidence suggests that the anesthetics effectively prevent the interaction between the electrochemically generated Fe(II) species and molecular oxygen, thereby hindering the subsequent formation of iron oxide-based polymers. This phenomenon can be rationalized by the strong spin decoherence induced by these halogenated molecules, a mechanism previously demonstrated to influence oxygen reduction reactions [8,9,10]. Eventually, the anesthetics influence, in terms of decrease in spin polarization/decoherence, on the ORR process can be viewed as antithetic to the role exerted by the CISS effect.

The present study serves as a model system for understanding how halogenated anesthetics can modulate oxygen interactions with metal centers. While the physiological mechanisms for these effects are still being investigated in vivo, our results from a controlled model system suggest a possible mechanistic link through anesthetic-induced spin decoherence.

## Figures and Tables

**Table 1:** Redox potential values for the Fe^II^/Fe^III^ couple of the Fe(acac)_3_ complex as determined via cyclic voltammetry.

	Scan Rate [mV/s]	$E_{red}$ [V]	$E_{ox}$ [V]	ΔE [mV]
Ar purged	50	−0.478	−0.386	93
	100	−0.513	−0.390	123
Saturate with O_2_ (in equilibrium with air)	50	−0.510	−0.365 ^†^	145
	100	−0.532	−0.370	163

^†^ Potential referring to a shoulder in the CV curve ($E_{ox}$) in Figure 2B, since no well-defined peak is detected.

## Data Availability

Dataset available on request from the authors.

## Supplementary Materials

The following supporting information can be downloaded at https://www.mdpi.com/article/10.3390/molecules30224341/s1, Figure S1: Blank solution CVs under O_2_; and Ar (1st and 2nd cycles), Figure S2: Fe(acac)_3_; CVs at 2 and 10 mM under Ar and O_2_; Figure S3: CVs at 2, 10, and 100 mM under Ar and O_2_; Figure S4: CVs with Sevoflurane (1st and 3rd cycles), Figure S5: CVs with Desflurane (1st and 3rd cycles), Figure S6: Electrode images after CV cycles (No anesthetic, Sevo, Desf), Figure S7: Raman spectra of films under Sevoflurane and Desflurane.
