# Supplementary material for: Sevoflurane and Desflurane Spin–Decoherence Effect on Fe(III)acetylacetonate Redox Process
Source: Molecules. 2025 Nov 10;30(22):4341. doi: 10.3390/molecules30224341 (PMC12654998; doi:10.3390/molecules30224341)
Supplement: Supplementary file 1 [file molecules-30-04341-s001.zip › molecules-3938454-supplementary.pdf]

Supporting Information

Sevoflurane and Desflurane Spin-decoherence Effect on Fe(III)acetylacetonate Redox Process.

Neha Kumari[1]†, Andrea Severini[2]†, Mauro Borghi[3], Monica Montecchi[1], Luca Pasquali[1,4,5], Elena Colombini[1], Gabriele Melegari[6], Alberto Barbieri[6], Enrico Giuliani[7], Massimo Innocenti[8,9], Fabrizio Roncaglia[2,10], Tapan Das Kumar[11], Claudio Fontanesi[1,8]*.

[1]Department of Engineering "Enzo Ferrari" (DIEF), Univ. of Modena, Via Vivarelli 10, 41125 Modena, Italy.
[2]Department of Chemical and Geological Science (DSCG), Univ. of Modena, Via Campi 103, 41125 Modena, Italy.
[3]Institute for Microelectronics (TU Wien), Gusshausstrasse 27–29, 1040 Vienna, Austria

[4] Department of Physics, University of Johannesburg, P.O. Box 524, Auckland Park 2006, South Africa.

[5] IOM-CNR, Strada Statale 14, Km. 163.5 in AREA Science Park, Basovizza, 34149 Trieste, Italy.

[6]Anaesthesia and Intensive Care, Azienda Ospedaliero Universitaria Modena, Via del Pozzo 71, 41211, Modena, Italy.

[7]NEURONGUARD, Via Ludovico Castelvetro, 19, 41124 Modena (MO), Italy.

[8]Department of Chemistry "Ugo Schiff", (DICU), Univ. of Firenze, Via Della Lastruccia, 41125 Sesto Fiorentino, Italy.
[9]National Interuniversity Consortium of Materials Science and Technology (INSTM), Via G. Giusti 9, 50121 Firenze (FI), Italy.
[10]Interdepartmental Centre H2-MORE, University of Modena and Reggio Emilia, Via Università 4, 41125 Modena, Italy.
[11]Department of Chemical and Biological Physics, Weizmann Institute of Science, Rehovot 7610001, Israel.

† Authors Neha Kumari and Andrea Severini contributed equally.

Table of Contents - Supporting Information

1. **Extended Cyclic Voltammetry Analysis**

   Figure S1. Blank solution CVs under O_2 and Ar (1st and 2nd cycles)

   Figure S2. $Fe(acac)_3$ CVs at 2 and 10 mM under Ar and O_2

   Figure S3. CVs at 2, 10, and 100 mM under Ar and O_2

   Figure S4. CVs with Sevoflurane (1st and 3rd cycles)

   Figure S5. CVs with Desflurane (1st and 3rd cycles)

2. **Influence of Sevoflurane and Desflurane on the Electrochemical Behavior of $Fe(acac)_3$**

   Peak data, molecular mechanism interpretation

3. **Electropolymerization and Raman Spectral Analysis**

   Figure S6. Electrode images after CV cycles (No anesthetic, Sevo, Desf)

   Figure S7. Raman spectra of films under Sevoflurane and Desflurane

1. Extended Cyclic Voltammetry Analysis

Please note that as specified in the main manuscript, The “base electrolyte solution” is indicated as “blank solution”, i.e. a solution prepared only with 0.1 M TBATFB (or TBAP) in MeCN. If the blank solution is purged with Ar no redox active species are present, thus the CV should be as “flat” as possible with the current close to zero. Indeed, in the 0 to -1.0 V potential window for the solution purged with Ar the current is quite close to 0 A: red line CV curves reported in Figure S1 A&B.

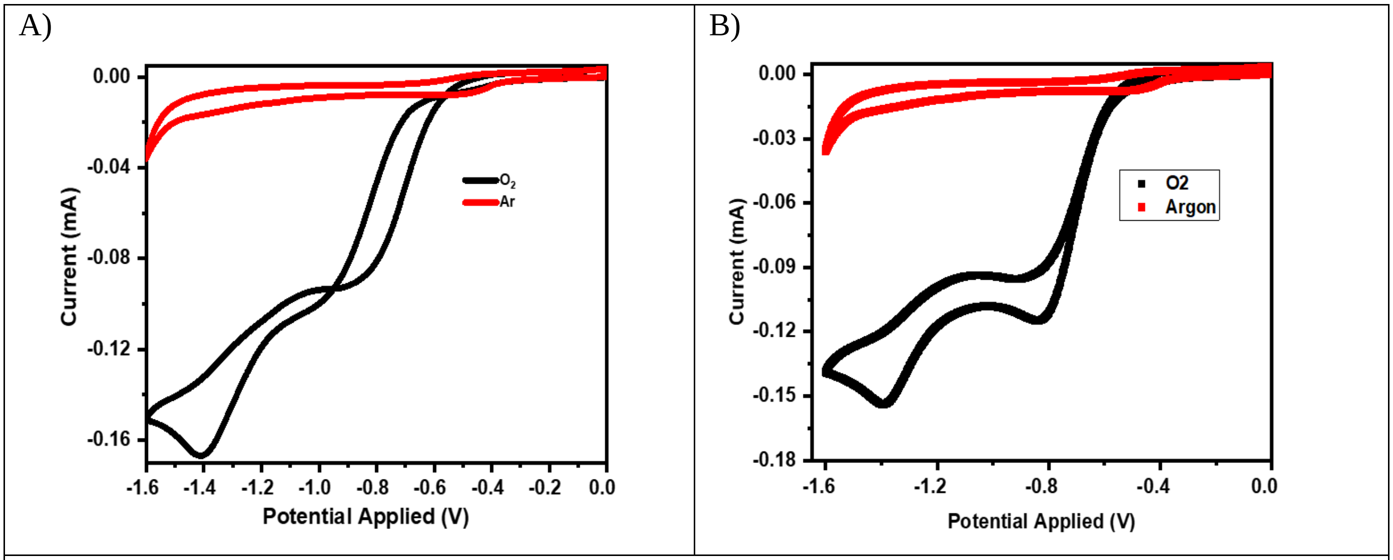

Figure S1. CV curves of Blank Solution containing 0.1 M TBATFB, MeCN solvent. Au, Pt, and Ag/AgCl/KClsat are the working, counter, and reference electrodes, respectively. A) 1st Cycle. B) 2nd Cycle. 50 mVs^{-1} is the potential scan rate.

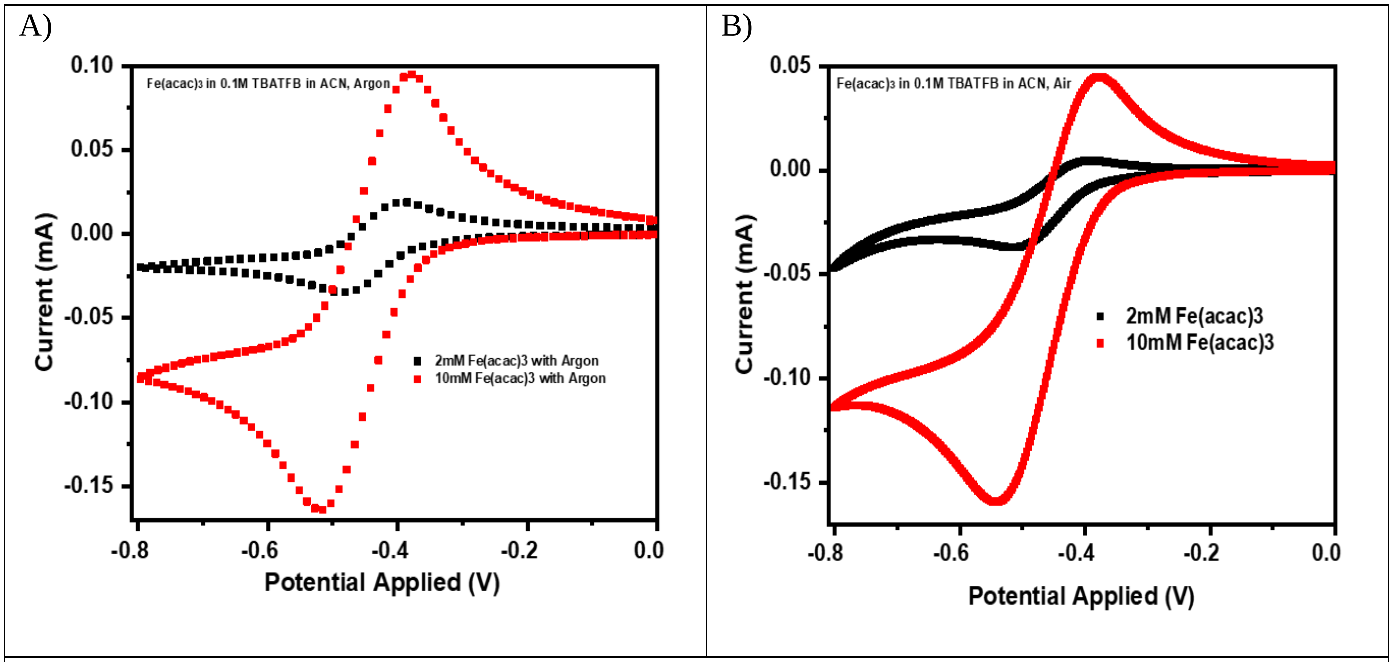

Figure S2. CV curves of 2 and 10 mM Fe(acac)3 in 0.1 M TBATFB, MeCN solvent. Over a potential window from 0.0 V to -0.8 V. Au, Pt, and Ag/AgCl/KClsat are the working, counter, and reference electrodes, respectively. A) Solution after 15 minutes, Ar purging. B) solution oxygen saturated at RT. 50 mVs^{-1} is the potential scan rate.

Figure S1 shows the cyclic voltammograms of the blank electrolyte solution (0.1 M TBATFB in MeCN) recorded under oxygen and argon atmospheres. These control scans demonstrate that the electrolyte itself is electrochemically inert within the studied potential window, confirming that the observed currents in $Fe(acac)_3$ solutions arise from redox-active species.

Figure S2 presents additional CV curves comparing 2 mM and 10 mM $Fe(acac)_3$ under argon (panel A) and oxygen-saturated conditions (panel B). The data illustrate the concentration-dependent electrochemical response and the clear influence of dissolved oxygen on reversibility and current intensity. Full discussion of these effects, including peak positions and mechanistic interpretation, is provided in the main manuscript (see Results & Discussion, Figure 1).

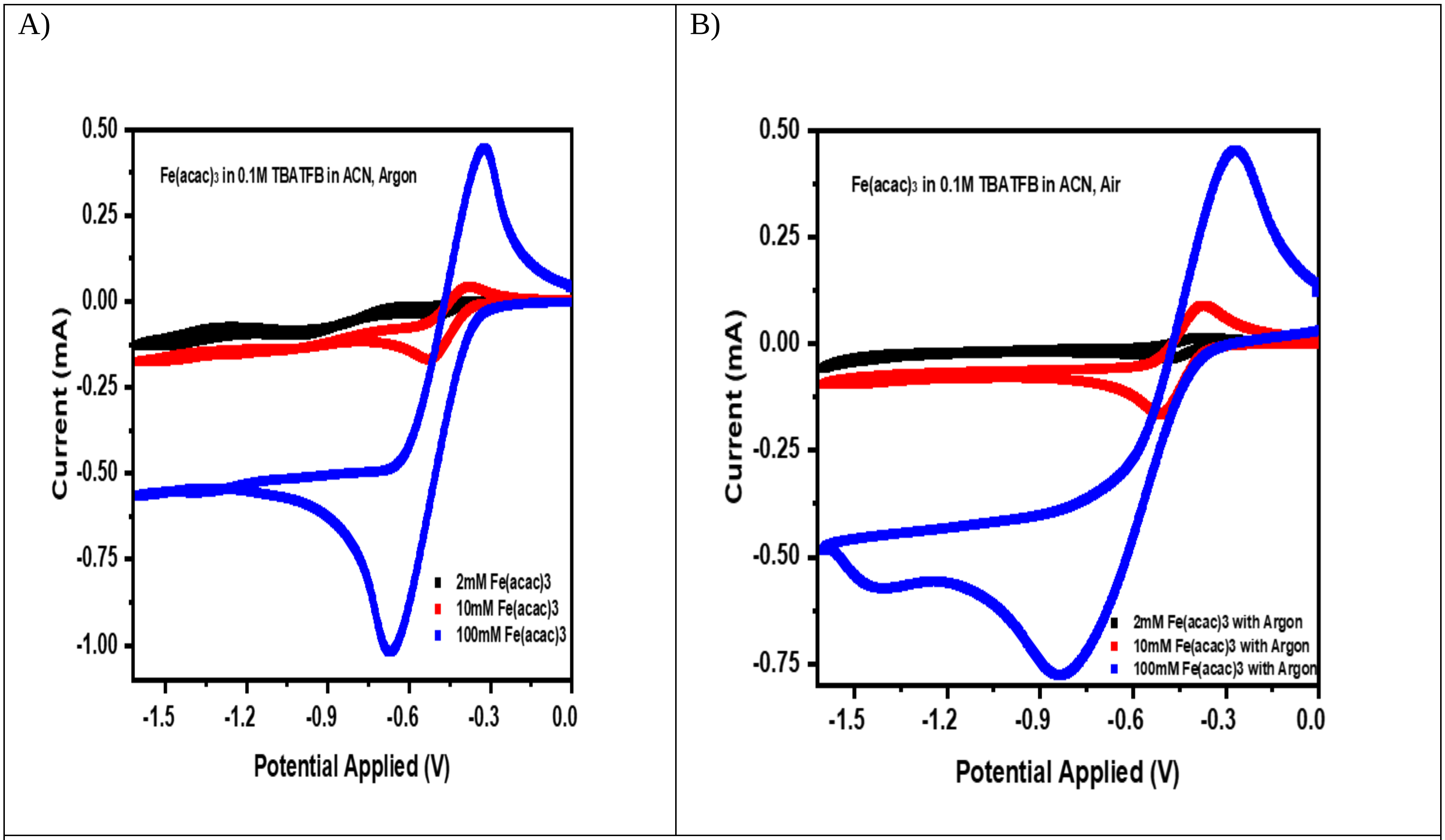

Figure S3. CV curves of 2, 10, and 100 mM Fe(acac)3 in 0.1 M TBATFB, MeCN solvent. Over a potential window from 0.0 V to -1.6 V, Au, Pt, and Ag/AgCl/KClsat are the working, counter, and reference electrodes, respectively. A) Solution after 15 minutes, Ar purging. B) solution oxygen saturated at RT. 50 mVs^{-1} is the potential scan rate.

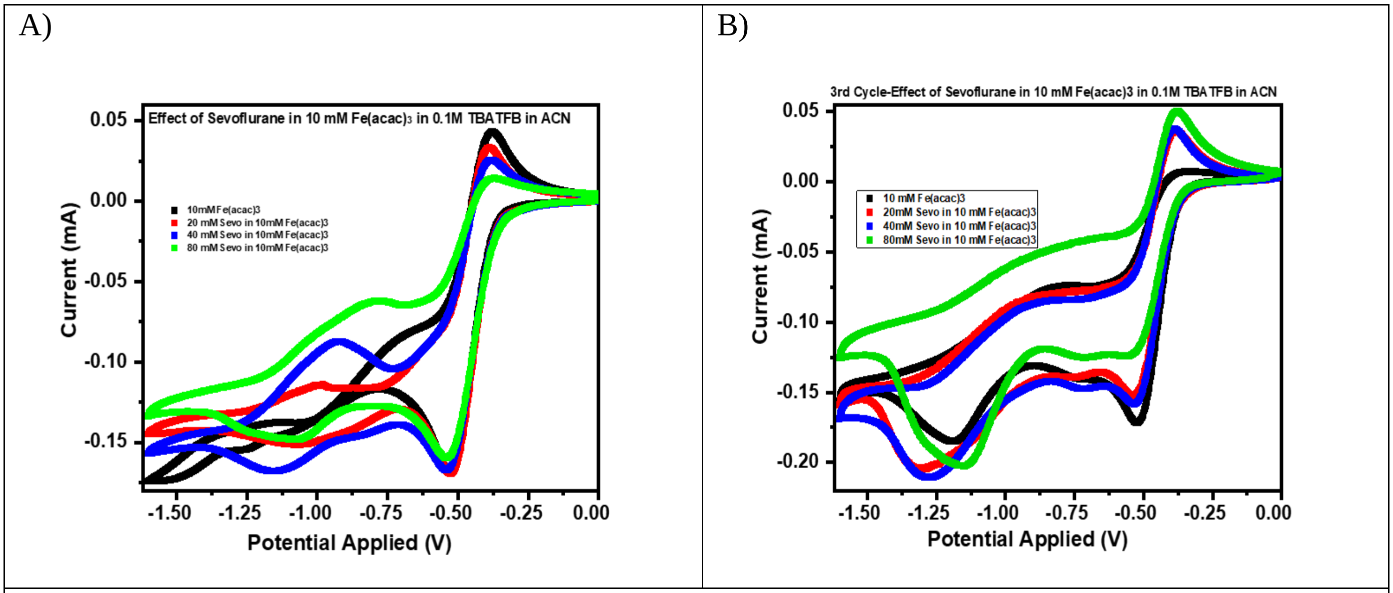

Figure S4. Oxygen saturated at RT, CV curves recorded in a 10 mM Fe(acac)3 in 0.1 M TBATFB, MeCN solvent solution. Au, Pt, and Ag/AgCl/KClsat are the working, counter, and reference electrodes, respectively. 50 mV s^{-1} is the potential scan rate. A) 1st Cycle, Black solid curve: 10 mM Fe(acac)3 solution. Red, Blue, Green solid curve: 10 mM Fe(acac)3 with the addition of 20, 40, and 80 mM sevoflurane. Data clearly show a systematic enhancement in current density upon increasing Sevoflurane concentrations. B) 3rd Cycle, Black solid curve: 10 mM Fe(acac)3 solution. Red, Blue, Green solid curve: 10 mM Fe(acac)3 with the addition of 20, 40 and 80 mM sevoflurane. Stabilization and enhancement of electrochemical activity with repeated cycling.

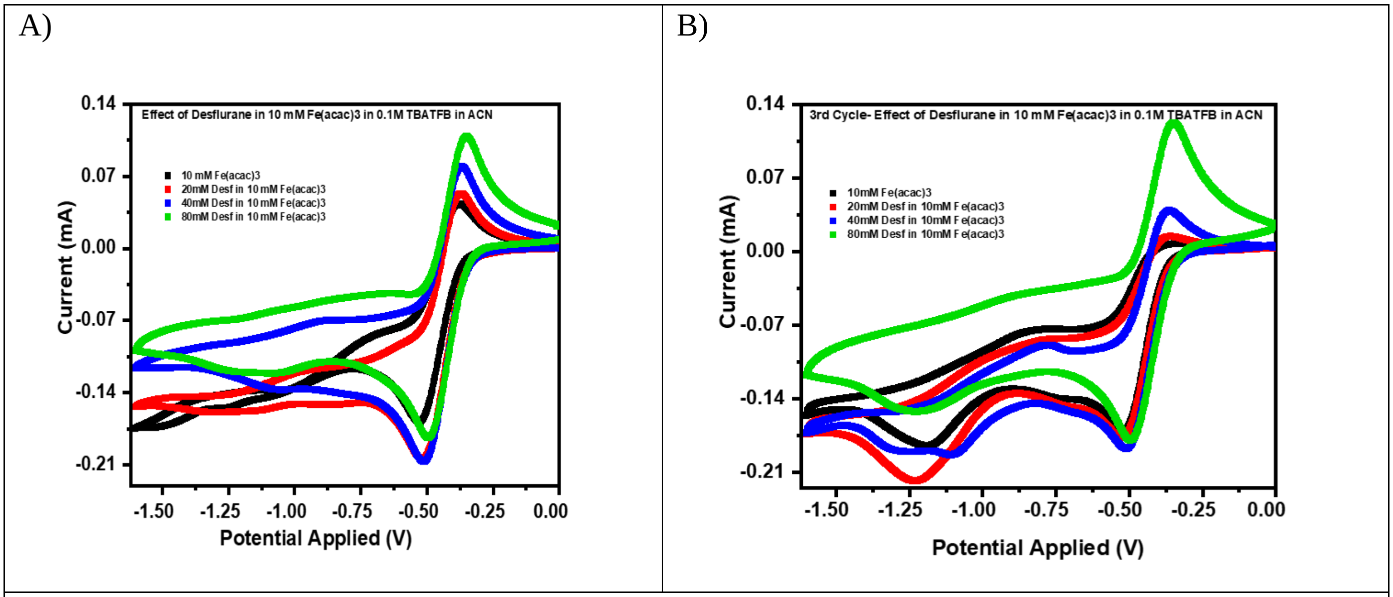

Figure S5. Oxygen saturated at RT, CV curves recorded in a 10 mM Fe(acac)3 in 0.1 M TBATFB, MeCN solvent solution. Au, Pt, and Ag/AgCl/KClsat are the working, counter, and reference electrodes, respectively. 50 mV s^{-1} is the potential scan rate. A) 1st Cycle, Black solid curve: 10 mM Fe(acac)3 solution. Red, Blue, Green solid curve: 10 mM Fe(acac)3 with the addition of 20, 40, and 80 mM Desflurane. B) 3rd Cycle, Black solid curve: 10 mM Fe(acac)3 solution. Red, Blue, Green solid curve: 10 mM Fe(acac)3 with the addition of 20, 40, and 80 mM Desflurane.

2. Influence of Sevoflurane and Desflurane on the Electrochemical Behavior of $Fe(acac)_3$

Figures S4 and S5 reveal that in the first Cycle, the cyclic voltammogram of the baseline solution (black curve) reveals clear cathodic and anodic peaks at approximately -0.53 V and -0.4 V, respectively, corresponding to the Fe(III)/Fe(II) redox couple. Upon the addition of Sevoflurane, the voltammetric response significantly changes. With increasing Sevoflurane concentration (20 mM, 40 mM, and 80 mM), a systematic increase in current density is observed, indicating enhanced electron transfer processes. These results suggest that Sevoflurane interacts with the $Fe(acac)_3$ redox system, possibly through coordination or interaction mechanisms, influencing the electrochemical characteristics and modifying the electrode surface dynamics. While in the third Cycle of the CV for the bare 10 mM $Fe(acac)_3$ solution (black trace) exhibits a quasi-reversible redox couple centered around -0.53 V (cathodic) and -0.4 V (anodic), attributed to the Fe(III)/Fe(II) redox transition. Upon the stepwise addition of Sevoflurane (20 mM - red, 40 mM - blue, and 80 mM - green), a significant increase in cathodic and anodic peak currents is observed by the third cycle, indicating enhanced redox activity and possible interactions [1] between $Fe(acac)_3$ and Sevoflurane. Notably, the third-cycle data show stabilized and amplified electrochemical features compared to earlier scans, suggesting that Sevoflurane facilitates the establishment of a more electroactive system upon repeated cycling, possibly through adsorption, coordination, or hydrogen-bonding mechanisms. These results confirm the reproducible and concentration-dependent effect of Sevoflurane on the electron transfer behavior of $Fe(acac)_3$.

Effect of Sevoflurane (80 mM) - Figure S4 A and B

- Cathodic peak (C_2): -0.53 V, –0.102 mA (-19 % current attenuation vs. Fe-only)
- Anodic peak (A_2): -0.35 V, +0.046 mA (+64 % current gain vs. Fe-only)

Sevoflurane (CF_3–CH(O)–CF_2H) is a moderately electron-withdrawing but weakly coordinating molecule. In MeCN, it forms a transient σ-hole/halogen-bond adduct with the Fe(III) centre (eq 1). The adduct slightly weakens back-bonding to the acetylacetonate ligands, lowering the Fe(III/II) current on the *forward* (reduction) sweep. On the reverse sweep, the adduct dissociates faster than the voltammetric timescale, increasing oxidation current.

$$[\mathrm{FeIII(acac)3}] + \mathrm{Sevo} \rightleftharpoons [\mathrm{FeIII(acac)3 \cdots Sevo}]$$

Electrochemically, the system behaves like an EC′ mechanism, in which the chemical release of Sevo ($k \approx 5\ s^{-1}$) regenerates free Fe(III), accounting for the 64% rise in A_2 without a significant shift in E_p.

Figures S4 and S5 reveal that in the first cycle, the black trace represents the CV of 10 mM $Fe(acac)_3$ alone, exhibiting a quasi-reversible redox couple centered at -0.53 V and -0.4 V, consistent with the Fe(III)/Fe(II) redox process. Upon incremental addition of Desflurane (20 mM - red, 40 mM - blue, 80 mM - green), a concentration-dependent enhancement in cathodic and anodic peak currents is observed. This indicates that Desflurane modifies the redox activity of $Fe(acac)_3$, possibly via weak coordination or solvent reorganization effects. Additionally, a slight shift in the peak potentials is noticeable, suggesting altered electron transfer kinetics or local solvent environment in the presence of the anesthetic. The overall CV profile confirms the interactive role of Desflurane in tuning the redox properties of the $Fe(acac)_3$ complex. While in the third Cycle, the black trace represents the baseline response of 10 mM $Fe(acac)_3$ alone, showing redox peaks associated with the Fe(III)/Fe(II) couple. Upon addition of Desflurane at increasing concentrations (20 mM - red, 40 mM - blue, 80 mM - green), a progressive enhancement in both cathodic and anodic peak currents is observed, along with slight peak broadening. These changes are particularly pronounced at 80 mM Desflurane, where the redox peaks shift slightly and the peak

current increases, indicating potential interaction or coordination between Desflurane and the $Fe(acac)_3$ complex. Notably, the effect remains consistent into the 3rd cycle, semi-stable electroactive species, potentially linked to spin coherence effects under repeated redox cycling[3]. or complex under repeated scanning. This supports the hypothesis of a weak but reproducible interaction between the halogenated anesthetic and the metal-ligand framework of $Fe(acac)_3$.

Effect of Desflurane (80 mM) - Figure S5 A and B

- Cathodic peak (C_3): - 0.54 V, -0.085 mA (- 32 % vs. Fe-only)
- Anodic peak (A_3): -0.34 V, +0.071 mA (+154 % vs. Fe-only)

Desflurane (CF_3-CHFO-CF_2-CHF_2) is more highly fluorinated and much stronger as an electron-withdrawing, weak Lewis base. It therefore gives a tighter halogen-bond (KHB ≈ 80 M^{-1}) to Fe(III), pushing more of the complex into the adducted form that is harder to reduce (larger cathodic current loss). The released $[FeII(acac)_3]^-$, however, is now stabilised by weak H-bonding from Desflurane, so its re-oxidation is faster, and A_3 nearly triples relative to the baseline.
A second, broad anodic shoulder at –0.26 V, +0.060 mA appears only with Desflurane; this is assigned to partial oxidation of a superoxide adduct:

$$[FeII(acac)_3]^- + O2 \rightarrow [FeIII(acac)_3\text{-}O_2\bullet^-] \quad (E \approx -0.27\ V)$$

The shoulder disappears under Ar (not shown), confirming its O_2 origin.

Comparative Electrochemical Behavior of Anesthetics

Comparing the third-cycle CV responses (Figure 2A) of Sevoflurane and Desflurane at high concentrations (80 mM). The black trace represents the baseline redox behavior of 10 mM $Fe(acac)_3$. Upon addition of 80 mM Sevoflurane (red curve) and 80 mM Desflurane (blue curve), a notable increase in peak current is observed, especially for the Desflurane-modified system. The Desflurane-containing solution shows a more pronounced cathodic current and slight anodic shift, suggesting enhanced electrochemical activity or interaction with the $Fe(acac)_3$ complex. This third-cycle data confirms that both anesthetics influence the redox behavior of $Fe(acac)_3$, with Desflurane exhibiting a stronger effect. This could be indicative of electron-donating/withdrawing interactions or coordination effects modulating the redox chemistry of the metal complex, suggesting a more potent interaction and modification of $Fe(acac)_3$'s redox behavior. This stronger interaction may be due to differences in electron affinity, steric hindrance, or coordination capability between the anesthetic agents and the $Fe(acac)_3$ complex.
Chemical reactions indicating anesthetic coordination effects could be proposed as:

$$Fe(acac)_3 + \text{Anesthetic} \rightleftharpoons Fe(acac)_3(\text{Anesthetic})$$

The anesthetic molecules stabilize the reduced Fe(II) state, shifting redox equilibria towards enhanced electrochemical activity and reactivity with dissolved oxygen:

$$Fe(acac)_3(Anesthetic) + O_2 + e^- \longrightarrow [Fe(acac)_3(Anesthetic)]\text{-}O_2^{-}\bullet$$

3. Electropolymerization and Raman Spectral Analysis

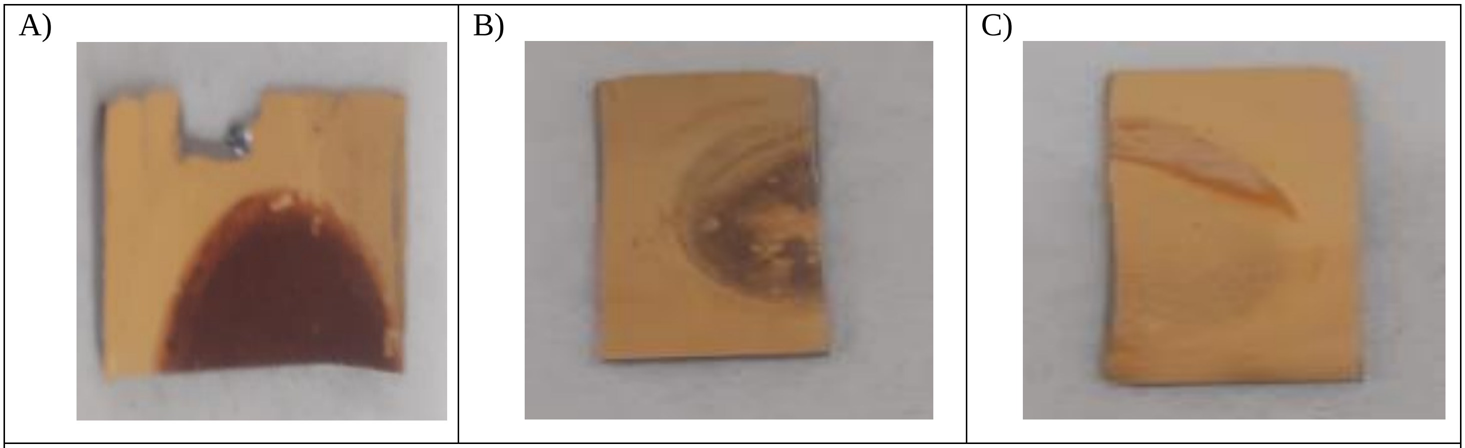

Figure S6. Photographs of the Au working electrodes after 68 CV cycles in oxygen-saturated 10 mM $Fe(acac)_3$ solutions (0.1 M TBATFB in MeCN, 50 mV s^{-1}). (A) No anesthetic added (reference), showing a thick reddish-brown polymeric deposit. (B) In the presence of 80 mM sevoflurane, the coating a thinner and patchier. (C) In the presence of 80 mM desflurane, there is negligible film deposition. These images highlight the strong inhibitory effect of volatile anesthetics on the growth of the polymeric Fe-based film.

The photographs in Figure S4 illustrate how volatile anesthetics alter the electrochemically driven film growth on gold electrodes. In the absence of anesthetics (panel A), repetitive cycling in oxygen-saturated $Fe(acac)_3$ solution produces a thick reddish-brown polymeric film due to the continuous reduction of Fe(III) to Fe(II) and its subsequent reaction with dissolved oxygen, generating reactive Fe–oxo species that deposit on the electrode. When sevoflurane (panel B) or desflurane (panel C) is present, these molecules interact weakly with the Fe center and dissolved oxygen, slowing down the electron-transfer cascade and suppressing the formation of reactive intermediates. As a result, film deposition is only partial with sevoflurane and is almost completely prevented with desflurane, consistent with their inhibitory effects on polymerization and the trends observed in the CV and spectroscopic data.

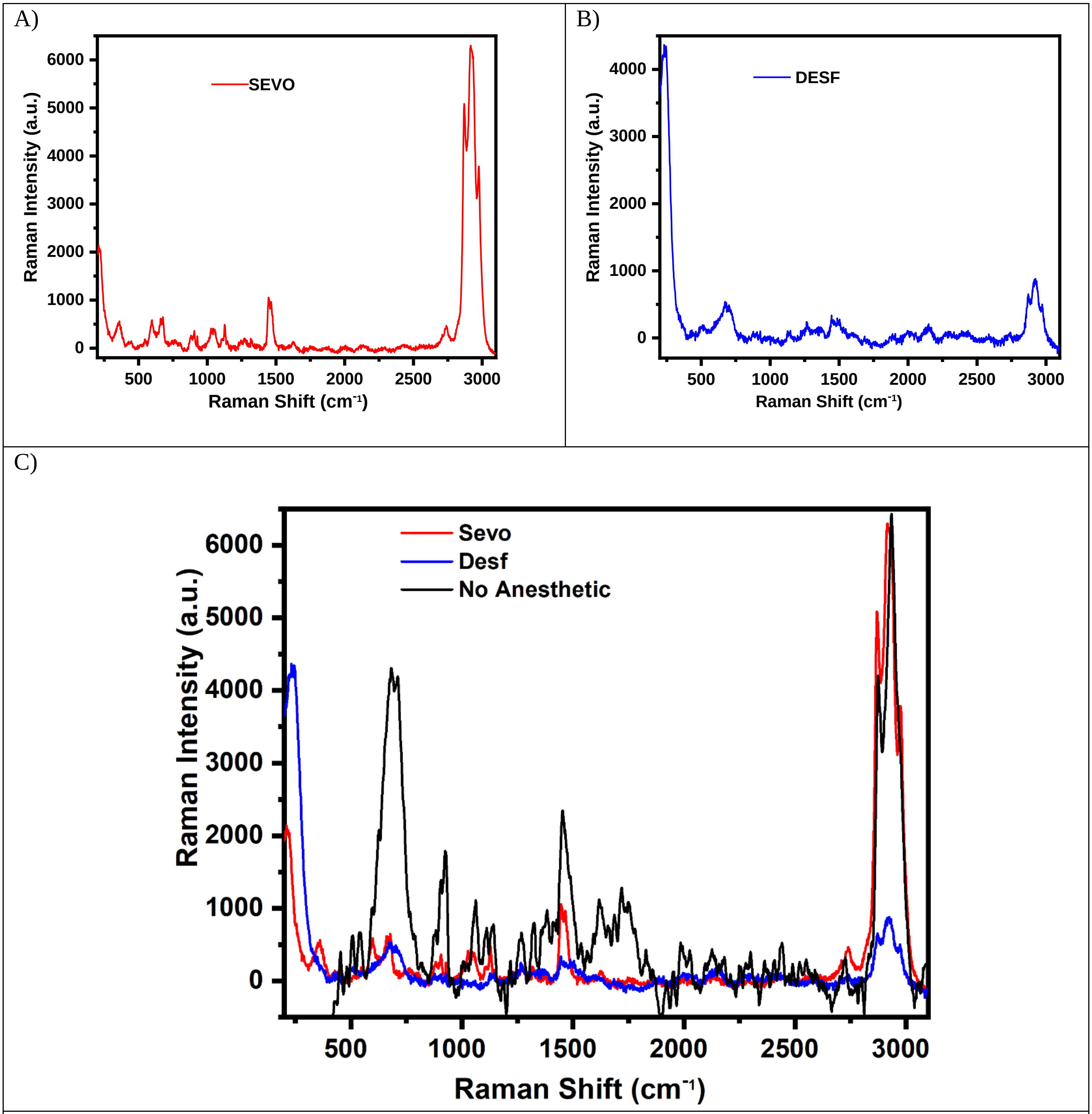

Figure S7. Raman spectra of polymeric films formed on a gold electrode after electropolymerization of 10 mM $Fe(acac)_3$ in 0.1 M TBATFB/CH_3CN, recorded under different conditions: (A) with 80 mM sevoflurane, (B) with 80 mM desflurane, and (C) comparison of spectra obtained without anesthetic (black), with sevoflurane (red), and with desflurane (blue). The films were prepared by cycling the potential from 0.0 to -1.6 V at 50 mV s^{-1} using a 1 cm diameter Au rod as the working electrode, Ag/AgCl as the reference electrode, and a Pt wire as the counter electrode.

Figure S7 provides complementary Raman spectra of the polymeric films formed on gold electrodes during the electropolymerization of 10 mM $Fe(acac)_3$ in 0.1 M TBATFB/CH_3CN in the presence of volatile anesthetics. While the main manuscript (Figure 3) presents the detailed analysis of the spectrum without anesthetics, these SI

data highlight how the addition of 80 mM sevoflurane (panel A) and 80 mM desflurane (panel B) modifies the vibrational profile of the resulting films. Panel C directly compares the three conditions, showing a significant attenuation of Fe–O and C=C band intensities as well as peak broadening in the presence of anesthetics. These effects are more pronounced for desflurane, which produces a more amorphous and less ordered film. This suppression of Raman features is consistent with the inhibition of polymer growth observed in cyclic voltammetry and with the changes in surface composition discussed in the main manuscript. Full peak assignments and detailed discussion of the spectral changes are provided in the main text (Results and Discussion, Figure 3).

Key vibrational peaks (Fe-O stretch at ~460 cm^{-1} and C=C stretch at ~1560 cm^{-1}) exhibit notable shifts and intensity variations, confirming structural alterations and the role of anesthetics. Detailed Raman Peak Assignments:

- 460 cm^{-1}: Fe–O stretching mode
- 1560 cm^{-1}: C=C stretching mode, indicating conjugation and ligand reorganization
- 2970 cm^{-1}: Aliphatic C–H stretching, influenced by anesthetic coordination

The differences observed in spectral features between solutions containing Sevoflurane and Desflurane suggest variable interaction strengths and distinct polymer morphologies induced by each anesthetic.
